# The unusual evolution of a simple bone cyst in the mandible: A case report

**DOI:** 10.4317/jced.50730

**Published:** 2012-04-01

**Authors:** Ignacio Velasco, Julio Cifuentes, Nelson Lobos, Felipe San Martín

**Affiliations:** 1 Professor, Department of Oral & Maxillofacial Surgery. Los Andes University, Chile; Resident, Department of Oral & Maxillofacial Surgery. Alemana Clinic, Chile; 2 Oral & Maxillofacial Surgeon, Department of Oral & Maxillofacial Surgery. Alemana Clinic, Chile; 3 Professor, Department of Oral & Maxillofacial Pathology. Los Andes University, Chile; 4 Resident, Department of Oral & Maxillofacial Surgery. Alemana Clinic, Chile

## Abstract

The simple bone cyst, as newly classified by WHO in 2005, is a lesion related to the jawbones. Therefore, it is not a cyst, since it is a cavity devoid of epithelial tissue. It is a rare pathology affecting the mandible more than the maxilla. Its onset occurs mainly during the first two decades of life, irrespective of sex. The purpose of our report is to exhibit the particular case of a 17-year-old male patient whose radiography showed an osteolytic lesion in his right mandibular body. Exploratory surgery and biopsy are performed showing a simple bone cyst. Since then, he is controlled through imaging studies, but presenting an atypical evolution, with its size increasing considerably within a 4-year follow-up.

** Key words:**Case report, simple bone cyst, hemorrhagic bone cyst, solitary bone cyst, idiophatic bone cyst, mandible.

## Introduction

A Simple Bone Cyst (SBC) is also known as solitary cyst, traumatic bone cyst, hemorrhagic bone cyst, unicameral bone cyst, and idiopathic bone cavity. According to the latest WHO classification for head and neck tumours, in 2005, it has been included among related lesions affecting jawbones, along with fibrous dysplasia and the central giant cell granuloma (1). This is a rare pathology accounting for only 1% of tumours and cysts in the maxillofacial region; by definition, though, this is not a cyst since it does not have an epithelial lining. In surgical exploration it is very often described as an empty cavity, but it may hold a hematic and/or serous content (2). This pathological entity is not unique to maxillary bones, and is 90% more prevalent in the metaphysis of long bones such as the humerus (65%) and femur (25%) (3).

The SBC is present more frequently within the first two decades of life although it can also be found in older age groups with no predilection for either sex (2,4). The mandible is the part that is almost exclusively affected -61% of the posterior part of the mandible between the canine and ramus (2,4,5); but, when the maxillary bone is affected, the more affected area is the anterior sector (2).

SBC is generally found accidentally during routine radiographic studies, rather than showing a characteristic symptomatology, and can cause mild pain in the area and tooth displacements (5). Pathological fracture is unusual, but it has been described in the literature (5), without generally compromising the vitality of any neighbouring teeth (4). A number of theories have been proposed relating its etiology and pathogenesis, such as a sequela of intraosseous hematoma, alterations in calcium metabolism, mild infectious conditions, local bone growth alteration, orthodontic treatment, venous obstruction, and a localized alteration of bone metabolism resulting in the development of an area of osteolysis (6). One of the most widely accepted theories is that, following a trauma, a vascular dysfunction arises leading to a post-hemorrhagic ischemia, and a bone necrosis is induced ending up in the development of an empty bone cavity (6). Despite this, none of these theories is conclusive, and it is believed that some of these lesions present a multifactorial etiology (2,6). Furthermore, in some of these reports, they have been linked to other pathologies such as fibrous dysplasia, and bone cement dysplasia (7,8).

In imaging, a SBC is generally shown as a radiolucent unilocular lesion of variable sizes with or without a bulging and thinning of the jaw cortices, with various conical or rounded shapes, its edges surrounding the teeth roots without causing external root resorption (4,9,10). The final diagnosis is, then, provided by a histopathologic study because, covering the cavity, there is no epithelial tissue (1). Instead, there can be a thin layer of connective tissue that appears to be covering the cavity (9-11). Additionally, small deposits of new bone formation, collagen deposits, hemosiderin and/or some scattered giant cells can be found (12).

The lesion has a good prognosis, usually healing within a year following surgical exploration; yet persistence may occur (5,13,14). Hence we herein present the unusual evolution of a SBC within 4 years of clinical and imaging follow-up.

## Case Report

**Figure 1:**
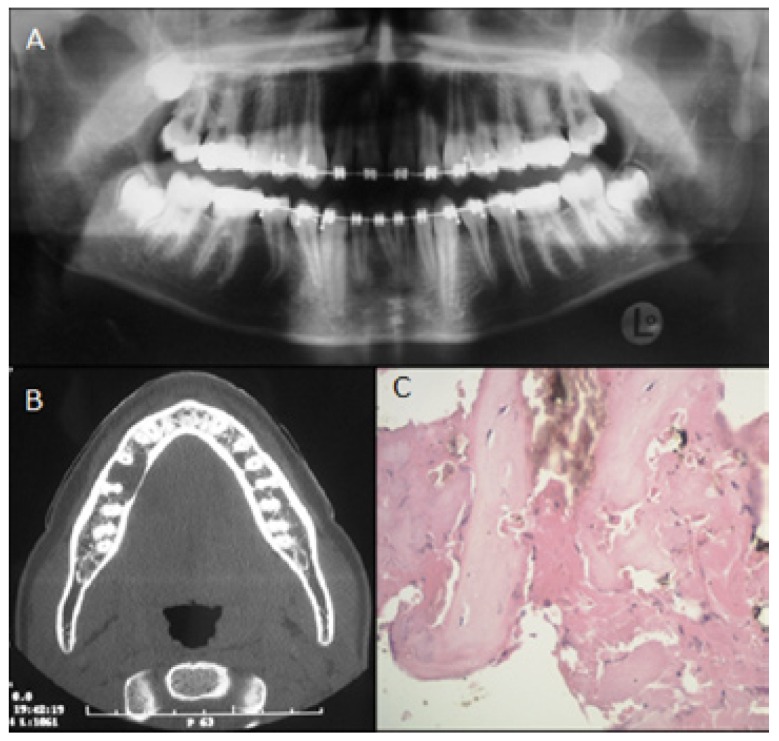
A.Panorex. B.CT: axial view. C.Histopathology: hematoxycilin-eosin stain, original magnification 400X.

A 17-year-old, Caucasian-race, male patient, without any clinically important morbidity background or facial trauma. Orthodontic treatment starts in the year 2005. During the year 2006, a control panorex is required which makes evident a radiolucent osteolytic lesion located in the right mandibular body between the apices of first and second premolar, with a rounded shape and well-defined limits (Fig. 1A). The patient consults with a private oral and maxillofacial surgery service (Santiago, Chile), where he is required to have an axial computed tomography (CT) taken for full information on the lesion, showing a mild lingual cortical bulging that appears to be remodelled and slightly thinned out though with no evidence of interruptions (Fig. 1B). The patient did not present any symptomatology, and the compromised teeth appeared to be vital. A biopsy of his lesion is then performed, resulting in a sample made up of pieces of vital lamellar bone tissue and recent hemorrhage areas (Fig. 1C), which –given the clinical background data- is altogether compatible with a SBC. A 1-to-2-year radiographic control is then suggested.

**Figure 2:**
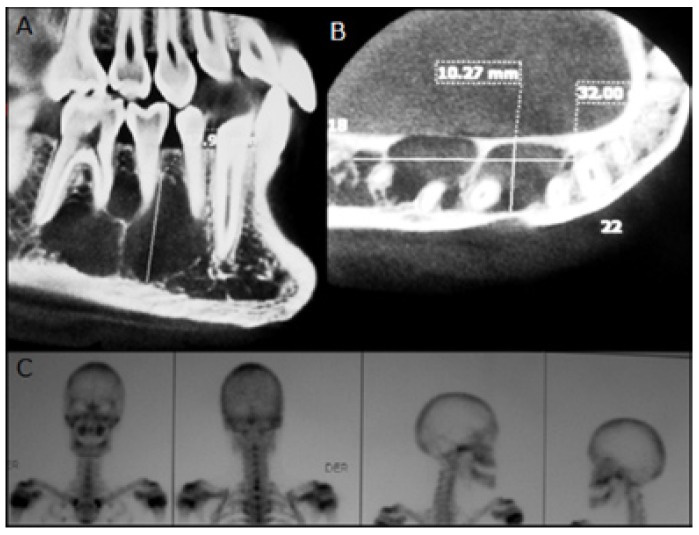
A-B. CB sagittal and axial view. C. bone scintigram.

One year after (2007), a control panorex shows a progression of the lesion with an increased antero-posterior size from right mandibular canine to first molar. Due to this progression, a Cone Beam (CB) is ordered to ascertain the real extent of the lesion, thus making evident an extensive hypodense lesion with defined and partially corticalized limits measuring 32 x 10,27 x 17,9 mm. The lesion presents evident loculations inside of it, with a lingual cortical strongly thinning out and without causing external root resorption of the compromised teeth (Fig. 2A, Fig. 2B). Even in the face of clinical and histopathologic SBC background data, the possibility of a more aggressive lesion with an odontogenic keratocystic tumour (OKT), ameloblastoma or other similar imaging features was not ruled out.

Early in 2008, a bone scintigraphy is performed intended to discard any proliferative lesion. The examination gave negative results with regard to some osteolytic lesion; no other treatments were ordered, and it was decided to control him by way of another CB within another 6 months (Fig. 2C). By mid 2008, then, another CB was performed, which did not show any significant variations from the last one.

**Figure 3:**
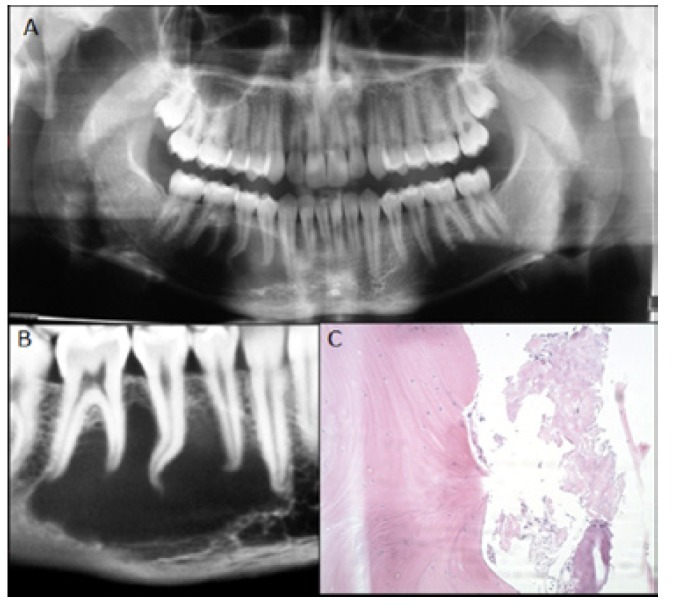
A.Panorex. B.CB: sagittal view. C.Histopathology: hematoxycilin-eosin stain, original magnification 100X.

In 2010, the patient consulted on his evolution at the Maxillofacial Surgery service of Alemana Clinic (Santiago, Chile). His extraoral and intraoral examination did not show any alterations. No evidence was found of adenopathy or paresthesia, and his mandibular mobility was normal; so were his mucosa and surrounding gums and teeth with reference to the lesion, with no presence of pathologies and vital (positive electric vitalometry). A CB is required to evaluate the extent of the lesion, which is found to have grown bigger compared to previous imaging studies made, covering from right mandibular canine to mesial of second molar, measuring 34 x 11.9 x 22.5 mm, without causing external root resorption (Fig. 3A,Fig. 3B).

Given the atypical lesion evolution, a new biopsy is programmed since the lesion appears to be similar to the OKT in the images. A complete blood count is requested along with blood biochemistry and plasma calcium levels, all within normal value ranges. The new surgical exploration for the new lesion biopsy showed an empty cavity with mild haematogenous content. Again, the result of this second biopsy shows the presence of vital lamellar bone tissue with recent hemorrhage areas, again confirming the diagnosed SBC (Fig. 3C). The evolution of the patient has been adequate, and he will be followed for not less than 3 years with clinical and radiographic examinations.

## Discussion

This case report describes a 17-year-old male patient with a unilocular lesion that is consistent with a SBC diagnosis, atypically changing its radiographic presentation to multilocular; yet, the SBC diagnosis is reconfirmed by the second biopsy. As reported by a great number of authors, SBC prevails in both sexes, with an average age of 18 years, is present in the posterior mandibular area, and is more prevalent among Caucasians (1,2,5).

SBC etiopathogenesis has failed to be widely investigated to date. Despite the great diversity of theories presented in related literature –irrespective of the skeletal location of the SBC— its etiology is not clear enough. Harnet et al. (6) have undertaken a review of the 3 prevailing theories: 1) an abnormal growth of the jawbone, 2) a process of tumour degeneration, and 3) a factor that triggers a bleeding trauma. The latter theory has been the most widely accepted hypothesis, which is based on the formation of an intramedullary hemorrhage, followed by a post-traumatic hematoma. The pressure of the hematoma brings on venous stasis leading to medullary necrosis and osteoclastic resorption due to decreased tissular pH (6). This theory could apply to the jawbone due to the multiple microtraumas undergone by the teeth and alveolar processes (6). A great number of authors have questioned this mechanism since there is no trauma history in over 50% of the cases reported. For this reason, it is thought to be a multifactorial etiology in a great number of cases (6,14).

SBC radiographic features often suggest the diagnosis, but this entity can be confused with a wide variety of odontogenic and nonodontogenic radiolucent lesions of the jawbones, as the unicystic ameloblastoma and OKT (4,11,12). Therefore, surgical exploration is needed to establish the correct diagnosis. Because at the time of surgery little tissue is obtained, the diagnosis is achieved in conjunction with the clinical, radiographic and histological features (11,12).

The SBC treatment of long bones is more aggressive and includes intralesional steroids injections with complete curettage of the osseous walls (3). Simple surgical exploration to establish the diagnosis is usually described in literature as sufficient therapy for jawbones lesions, and recurrence or persistence of the lesion is unusual (2,4,5,15). However, with the exception of a few reports, little attention has been given to the jawbone SBC prognosis. Recurrence rates as low as 1.3% and 1.6% (15) have been reported in review articles, but these studies included cases with no description of treatment results. Suei et al. (14) made a review of 132 own cases published in the literature, showing a recurrence of 26%, with all these cases followed up until their healing or recurrence, and with recurrence rates being very similar to extracranial SBC studies (29%)(3). The relationship between SBC surgical procedure and prognosis has never been established. However, it is stated in the literature that SBC is healed following a simple surgical exploration or bone curettage (5). Suei et al. (14) show in their results that SBCs undergoing this treatment have the highest recurrence rates (>20%), whereas the prognosis improved when the treatment was fenestration or packing the cavity.

The average time from surgery to complete healing ranges from 8.5 to 21.5 months, depending on the treatment performed (5,13,14). For this reason, during follow-ups, clinical-radiographic evaluation is important if healing or recurrence is to be established, since recurrence is described to occur 2 years to 2 years and 5 months following surgery (14). Therefore, healing is on average faster than recurrence by 1 year, speculating that initial bone regeneration following surgery is faster and more noticeable in cases of healing than recurrence. As a result, healing or recurrence can be predicted at early stages, following surgery, by way of radiographic evaluation (14); as happened with our report showing recurrence and change in its radiographic presentation, resembling other jawbone tumoral entities such as keratocystic odontogenic tumor or ameloblastoma.

Some authors suggest that the first radiographic control should take place between 12 and 17 months following surgery, because it is during this time when healing is more clearly confirmed (14). Recurring lesions usually grow slowly and do not generate any frequent complications such as pain or a pathological fracture. Therefore, an early identification of recurrences is not particularly helpful. After the postoperative evaluation, clinical-radiographic evaluations at yearly intervals are recommended, as sufficient since most healing or recurrence cases are confirmed during the first or second postoperative examination.

In conclusion, SBC is a rare jawbone pathology with an unclear etiology. It is generally associated to a good prognosis and a low rate of recurrence. Yet, we have reported an unusual mandibular SBC recurrence and evolution. An adequate postoperative follow-up is recommended for this pathology for at least 3 years at yearly intervals, along with the corresponding radiographic control in order to properly establish its healing or recurrence.
